# AI Chatbot Design during an Epidemic like the Novel Coronavirus

**DOI:** 10.3390/healthcare8020154

**Published:** 2020-06-03

**Authors:** Gopi Battineni, Nalini Chintalapudi, Francesco Amenta

**Affiliations:** 1 E-Health and Telemedicine Centre, School of Pharmaceutical Sciences and Health Products, University of Camerino, 62032 Camerino, Italy; nalini.chintalapudi@unicam.it (N.C.); francesco.amenta@unicam.it (F.A.); 2 Studies and Research Department, International Medical Radio Center Foundation (C.I.R.M.), 00144 Rome, Italy

**Keywords:** nCOV-19, AI, chat bots, artificial intelligence markup language (AIML), functionalities

## Abstract

Since the discovery of the Coronavirus (nCOV-19), it has become a global pandemic. At the same time, it has been a great challenge to hospitals or healthcare staff to manage the flow of the high number of cases. Especially in remote areas, it is becoming more difficult to consult a medical specialist when the immediate hit of the epidemic has occurred. Thus, it becomes obvious that if effectively designed and deployed chatbot can help patients living in remote areas by promoting preventive measures, virus updates, and reducing psychological damage caused by isolation and fear. This study presents the design of a sophisticated artificial intelligence (AI) chatbot for the purpose of diagnostic evaluation and recommending immediate measures when patients are exposed to nCOV-19. In addition, presenting a virtual assistant can also measure the infection severity and connects with registered doctors when symptoms become serious.

## 1. Introduction

After the rise of the web and mobile apps, virtual chatbot applications are the latest inventions of digital design [1,2]. These applications are well known for automatic conversational agents that run on computer programming or a kind of artificial intelligence (AI) interaction between the users and machines with the intervention of natural language processing (NLP) [3]. Chatbots are potentially referred to as the most promising and advanced form of human-machine interactions [1,4]. Eventually, these virtual agents are getting involved in the main global sectors such as healthcare, banking, education, agriculture, etc. [4].

The healthcare sector is closely associated with human interaction, and it seems counterintuitive that conversational AI applications like chatbots are more prevalent [5]. Hospital administrators are spending their day in appointment scheduling and answering routine questions of patients. Continuing or repeating the same actions and words is neither necessary nor productive. Such jobs can be easily done with bot applications. It is obvious that patient feedback assessments are also possible by collecting user responses to maintain good patient flow. In the occurrence of serious pandemics like novel Coronavirus (nCOV-19), health bots are beneficial as a supplement to personal clinical care or immediate medications.

After nCOV-19 spread beyond China, it spread globally at a rapid pace and about six million cases have been confirmed [6]. Because of continuous patient flow, it has been a great challenge for national governments to supply enough medical specialists, resources, and equipment to hospitals or healthcare centers. Therefore, we designed an AI medical chatbot to ease the burden of healthcare systems by identifying infection severity. In addition, it has all the necessary preventative measures including interactions with live doctors.

There are some established nCOV-19 virtual agents integrated with messenger applications. The World Health Organization (WHO) has launched a dedicated messenger app in seven languages to keep the public safe from coronavirus infections [7]. Likewise, the German government developed a ‘fight COVID messenger bot’ [8], the Bangladesh-based SAJIDA Foundation developed an nCOV-19 information bot with a symptom checker and explanations of preventive measures [9]. While in India, the Aarogya Setu mobile app has been recently developed to create awareness of nCOV-19 with the parallel connection of a chatbot [10]. However, all of these bots are serving as medical consultants of the coronavirus, and none of them highlight the issues concerning remote patients in terms of the pandemic.

Therefore, our proposed personal health chatbot for rural patients will act as a medical consultant, and also provides simple and relevant measures of not being infected by nCOV-19. Another advantage of this bot includes 24/7 accessibility and assesses the patient’s condition in a more human-like way. Due to the built-in backend logic function, it will detect the virus’s intensity and provide live interactions with doctors in the handling of dangerous conditions.

## 2. Framework and Functionality

The designed bot can handle user requests and identify message patterns with an artificial intelligence markup language (AIML). AIML is an XML-based markup dialect to create natural language software agents and gives the real human interactive experience to users [11]. Depending on user responses, AIML logic retrieves symptomatic keywords to assess the existing user medical conditions. Ultimately, we aim to make sure the user feels like they are having a conversation with a health specialist.

**Figure 1:**
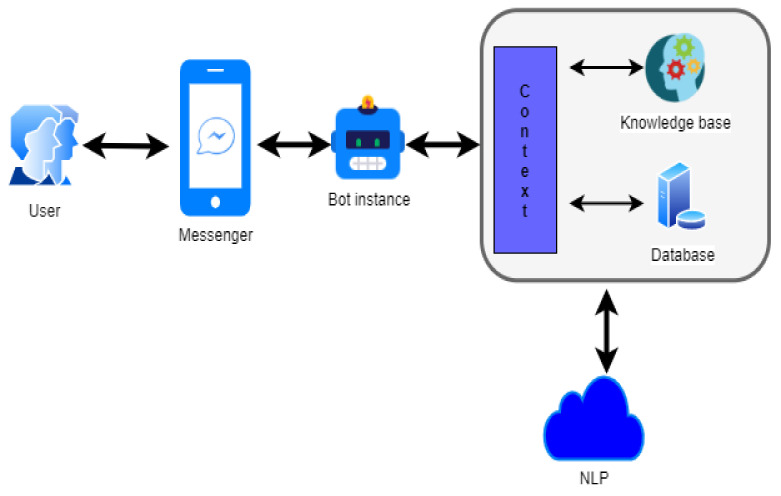
The designed framework of AI chatbot functionality.

The functionality of chatbot is defined in two ways: request analysis or return response. At first, chatbot evaluates the severity of the virus through feedback from a predefined questionnaire. Simultaneously, if the user fails to acknowledge precise answers, the bot will fail to provide the correct response. In its response return, after the evaluation of a patient’s condition, the chatbot provides an obvious response in the form of either generic text or text retrieved from the knowledge base response (Figure 1).

A render question could help chatbot precisely understand the user’s request. To achieve the intended response, the fusion of AI technology with natural language programming (NLP) has been done, because NLP is a helpful technology to draw the relationship between natural language understanding and decides complementary response outcomes [12].

The knowledge base is focused on user message response during the initiation of conversation—it should be natural at responding with a suitable back-end logic. The idea behind this is to identify preliminary symptoms of nCOV-19 from the user location. Thereafter, it will display whether the user is likely to be infected or not.

**Figure 2:**
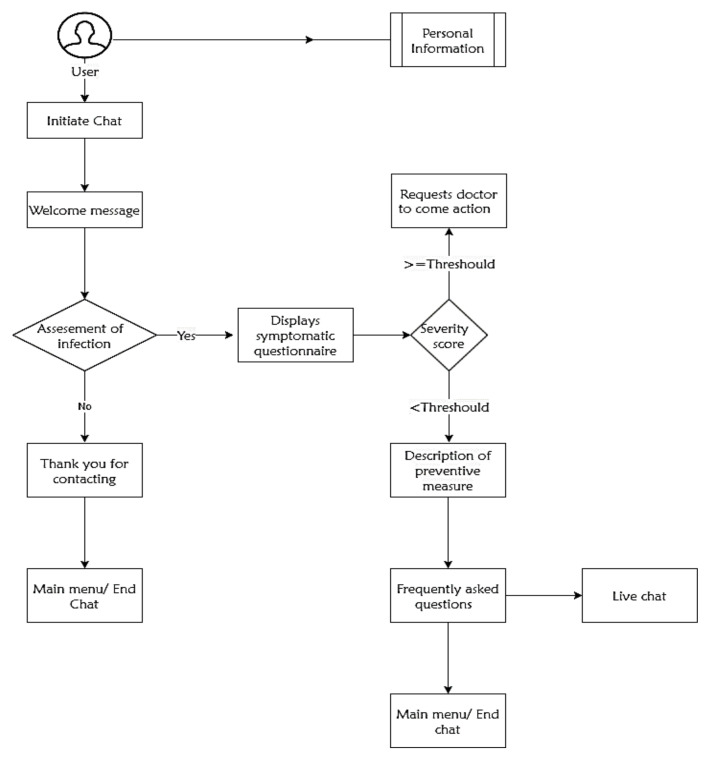
Working functionality of the developed chatbot.

After the user initiating the chat session, the bot engine starts questioning the person regarding symptomatic information. Once it has all of the necessary details, it finds the virus severity percentage that the user experienced and acts accordingly, by either making contact with health specialists or provides information regarding immediate preventive measures. Figure 2 depicts the systematic functionality of the proposed chatbot application.

## 3. Bot Design Schemes

### 3.1. AIML Component Design

The AIML works according to stimulus-response methods and provides simple dialogue modeling. As mentioned, it is an XML-based markup language with a tag basis. These tags are identifiers that insert commands and make code snippets in the chatbots. AIML defines data object classes as objects responsible for modeling patterns in conversation. The general format of AIML command/tag/objects has a structure as
*<Command>* *List of parameters* *</command>*

With the integration of AIML in our chatbot, it can detect patterns from user messages and provides precise and meaningful answers. Our chatbot also figures out if the user wants to check the infection status or just wants to know basic measures and symptomatic behavior of the nCOV-19 virus.

#### 3.1.1. Pattern Recognition with Snippets

Given that a chatbot should understand the patterns of user’s requests with predefined tags of the AIML component. In AIML, a predefined tag pattern (<pattern>) helps chatbot in recognition of virus symptoms and if it matches, the particular category of the questionnaire could be displayed. For instance, if the user suspects they have been infected, the following sample patterns with snippets could proceed as follows [13]:
*<Pattern> I am getting cough since three days </pattern>* *<Pattern> My body temperature is high </pattern>* *<Pattern> I shared bed with infected patient </pattern>* *<Pattern> I had travel history of migration in the last two weeks </pattern>*

In such the above-mentioned patterns, our chatbot looks for possible cases of being infected or if it crosses the threshold value immediately, it will connect to the health specialists.

#### 3.1.2. Dataset and Threshold Values Assessment

Any chatbot should be natural while responding to user responses and it needs to have a clear dataset and sustainable backend logic for outcome generation. At the Telemedicine department at the University of Camerino, we have developed a ten-question basic symptomatic questionnaire of COVID-19. This entire questionnaire is made of simple Yes/No type questions. If the user answered ‘Yes’, then the bot assigns the score of one, and if the user answered ‘No’, the bot gives a score of zero. The chatbot expects to use AIML logic to reply to user responses and sustain the input that could feed the machine. Table 1 presents the initial symptomatic inquiries that the chatbot can identify whether the user is infected or not.

Once the engine retrieves the user feedback for the given questionnaire, it will assess the symptomatic response score-defined AIML technique. We set a threshold value of AIML logic at ‘seven’, and if bot accumulated response value crosses this threshold limit, then the user will be connected directly to the doctor. Simultaneously, the bot will also anticipate immediate preventative measures to avoid direct contact with others.

When the bot engine feels the severity of the user’s symptoms has reached the threshold, it automatically alerts the users to contact health specialists immediately. In this scenario, chatbot requests a doctor to get in touch with the user with useful clinical information. To trigger this procedure, the pandemic severity score has to touch or cross the threshold level [14].

$$\mathrm{The}\;\mathrm{threshold}\;\mathrm{value}\;\mathrm{can}\;\mathrm{be}\;\mathrm{calculated}\;\mathrm{by}\;H=\frac{\sum \left(\mathrm{Score}\right)}{\mathrm{Threshold value}}$$

Here, H is the decision-making parameter that is used to check the threshold level, $\sum \left(\mathrm{Score}\right)$ is a total score of symptomatic information from user input, and the threshold value maximum limit of chatbot deciding if the user can handle the level of the condition. If H ≥ 1, then chatbot triggers the infection severity and connects to the doctor immediately.

#### 3.1.3. AIML Script for the Developed Bot

Initially, we created a project folder with two directories called *aiml_scripts* for AIML scripts and *configs*, cd into the project given folder. We created an AIML script called userpandemic_chat.aiml and placed it into the aiml_scripts folder with the following content:
*<aiml version="1.01" encoding="UTF-8">* *<!—user pandemic_chat.aiml -->* *<category>**<pattern>hello</pattern>**<template>**<random>**<li>hi (user name), I am a virtual health assistant to help you to identify whether you infected with NCOV-19 or not? You would like to know please press ‘yes’ or for preventive information press ‘no’</li>**<li>yes</li>**<li>thank you for the response, please respond all the questions that displayed here</li>**</random>**</template>**</category>*
*<category>**<Pattern>What is your gender? (Male/Female) </pattern>**<template>* *<li> male</li>**<li> great, what’s your age (20-35), (36-55), (55-75), >75 </li>* *<li> 36-55 </li>**<li>are you face any of following symptoms (cough/ Fever/Breath difficulties)</li>**<li>None</li>**<li>Nice, have you ever had any of the following (heart or lung diseases/hypertension/None of the above)</li>* *<li>None of the above </li>* *<li>it seems that your non infective member on board, however please follow the preventive measures that suggested and don’t hesitate contact us any time </li>* *<li>preventive measures document</li>* *<li>good day</li>**</random>**</template>**</category>* *</aiml>*

### 3.2. Engine Design

The conversational agent engine allows the chatbot to identify patient preferences, problem areas, and patient conditions. It identifies the behavioral patterns and autonomously learns from the conversation. The chatbot engine is a principal back-end logic that validates input using the method from Web API. After successful identification of infection severity, the engine maintains two different methods, such as contact with the doctor and immediate follow-up of household preventive measures. Each symptom defines with an integer variable to assess the seriousness score. Once all the symptomatic information is collected, the bot triggers a specialist consultation. The engine prepares a solution in the XML format for this action. AIML uses <template> and <sraix> tags to parse the XML feedback and user display.

### 3.3. Quality Evolution and Comparison

We considered ten different functional aspects of existing individual bots of nCOV-19 that were included in this study to evaluate each performance (Table 2). Primarily, each bot examines the visual look and implementation form on the website and the speech synthesis units. Simultaneously, to evaluate the knowledge base of reliably established bots, a similar line of questioning was asked for each virtual assistant. This enables us to understand language skills, conversation abilities, and text context of chatbots.

In addition, we explore other features like personality traits, personalization, and emergency responses with possible user ratings. To assess the quality components of each bot, we used a standard measurement tool rating of 1 to 5 with denotations like 1: very poor, 2: poor, 3: average, 4: satisfied, 5: excellent. Transparent and feasible measures are used, as there is no need to include advanced statistical analysis [15]. Scores of each functionality were given based on user feedback and online patient ratings. In the end, we consider the average score of ten functionalities to gain quality insight into the mentioned nCOV-19 chatbots.

From Table 1, it is clear that present bots have some functional limitations. We considered all of these problems while developing our new conversational agent. The WHO launched bot (4.0), and the Indian bot application Arogya Setu (4.05) had satisfactory ratings, followed by the other two bots, such as Fight nCOV-19 from Germany (3.7), and the SAJIDA corona bot from Bangladesh (3.55).

## 4. Discussion

Artificial intelligence consists of a heterogeneous set of techniques and methods for building intelligent systems with cognitive capabilities of recognizing, classifying, reasoning, diagnosing, or having at least some of these properties [16,17]. A list (incomplete) of the main problems covered by this discipline includes automatic reasoning, planning, diagnosis and automatic classification, understanding of natural language, learning, and robotics.

NLP is an artificial intelligence tool that is well-suited for clinical diagnostic issues and the development of symptomatic description pathways when in need of private medical care. It will be started by identifying the most common patient pathologies who live in remote areas like mountains, forests, and slums [18]. Because of the nonavailability of nCOV-19 clinical information from medical experts, our designed conversational agent with an elaborating questionnaire could enable us to address general questions. The questionnaire threshold outcome is a simple statistical test that represents the origin of developing the AI system symptomatology section.

In this study, we randomly selected four international chatbots for nCOV-19, including three messenger bots and one Indian application with the built-in chat feature. A comparison of each application was done with ten relevant functional aspects mentioned in Table 1. Each bot had some limitations and two of them only reached satisfactory outcome ratings. To overcome this, we designed a sophisticated chatbot application that surpassed the mentioned bots.

During implementation, the appearance of a chatbot would play a vital role in measuring quality standards. The bot’s visual look resembles the targeted users and enhances their involvement or desire to initiate a chat session. The adopted three messenger bots had an average visual quality, including graphics of a low quality. However, we maintain a good visual look to attract more users, and the knowledge base stores patient symptomatic information.

Speech synthesis units have a special feature of converting written text into a synthetic speech. We felt that no existing chatbot possessed this function with a unique custom voice coupled with a shutdown option. Conversational ability, personalization, and personality traits will be vital to identify symptomatic behavior of the nCOV-19 pandemic. Single embedded links can present preventive measures, and these links work as phrased tag words to the user’s input for ongoing conversation.

In an emergency, the chatbot provides patient location, symptomatic data, and the infection severity score to a doctor or healthcare organization’s automatic voice message alert. This data is then compared with the chatbot clinical knowledge base and delivers preventive actions to the local user. Similarly, it offers a chance to speak with a doctor 24/7 with a live chat feature; the user can feel confident in sharing their personal information of mental health or insight symptomatic behavior of the nCOV-19 virus.

## 5. Conclusions

Our idea behind this study is to present sophisticated AI medical chatbots for users, especially during unknown pandemics like nCOV-19. The presented AI chatbot will have a large impact on patient life during serious epidemics. It would provide the advantage of putting access to virtual doctors into their hands. We bring health specialists and professionals into our platform to feed medical information into a bot engine, also to the availability of every user whenever the possibility of infection is detected.

At present, the proposed chatbot is in the design phase, which will be followed up by total design into code soon, with plans to launch this app in the next few months. Initially, we would like to release the basic version soon, especially in the context of the severity of the present pandemic of the novel coronavirus. We are developing this chatbot engine in Python, and Watson as the AIML platform. After releasing this bot into the market, based on user feedback, further updates will be possible for at least 3–4 months. Once this COVID-19 pandemic is over, we plan to reuse this conversational agent and make it compatible with other epidemics or other services with individual APIs or relevant datasets.

## Figures and Tables

**Table 1:** Symptomatic inquiry dataset.

No.	Question	Type
1.	Do you have any kind of diabetes?	Yes/No
2.	Have you or your family been infected with nCOV-19?	Yes/No
3.	Do you know people in your immediate social environment who are or have been infected with the novel coronavirus?	Yes/No
4.	Are you closely interacting with other infected people in your region?	Yes/No
5.	Is your travel history associated with infected countries in the last two months?	Yes/No
6.	Have you used a bed or other premises previously used by someone who was infected by coronavirus?	Yes/No
7.	Have you been in contact with a suspected case-patient of COVID-19?	Yes/No
8.	Any shortness of breath?	Yes/No
9.	Are you facing a sudden rise in body temperature?	Yes/No
10.	Are you coughing often nowadays?	Yes/No

**Table 2:** Performance evolution and comparison of NCOV-19 bots (1: poor and 5: very satisfied).

Functionality	WHO nCOV-19 Launched Bot	Fight COVID Messenger Bot by Germany	SAJIDA Corona Bot Application in Bangladesh	Aarogya Setu in India	Our Bot
Visual Look	3	3	3	4.5	4.5
Form of Implementation on the Website	3.5	3.5	4	3.5	5
Knowledge Base	4.5	4	4	4.5	5
Speech Synthesis Unit	3	3.5	3	3	4.5
Knowledge presentation	5	4.5	4.5	4.5	5
Conversational Abilities, Language Skills and Context Sensitivity	4.5	3.5	4	4	4.5
Personalization	4	3.5	3	4	5
Personality Traits	4.5	4.5	4	4.5	4.5
Emergency Responses in Unexpected	4.5	4	3	4	5
Situations					
Possibility of Rating Chatbot	3.5	3	3	4	5
Overall Average	4	3.7	3.55	4.05	4.8
